# Density-independent population projection trajectories of chromosome-substituted lines resistant and susceptible to organophosphate insecticides in *Drosophila melanogaster*

**DOI:** 10.1186/1471-2156-5-31

**Published:** 2004-11-24

**Authors:** Takahiro Miyo, Brian Charlesworth

**Affiliations:** 1 Institute of Evolutionary Biology, School of Biological Sciences, University of Edinburgh, Edinburgh EH9 3JT, UK

## Abstract

**Background:**

Seasonal fluctuations in susceptibility to organophosphate insecticides were observed in the Katsunuma population of *Drosophila melanogaster *for two consecutive years; susceptibility to three organophosphates tended to increase in the fall. To examine the hypothesis that variation in fitness among resistant and susceptible genotypes could trigger the change of genetic constitution within the fall population, we investigated density-independent population projection trajectories starting from single adult females with characteristics of chromosome-substituted lines resistant and susceptible to the three organophosphates.

**Results:**

Density-independent population projection trajectories, expressed as the ratios of the number of each chromosome-substituted line to that of line SSS, for which all chromosomes were derived from the susceptible line, showed significant declines in numbers with time for all the resistant chromosome-substituted lines.

**Conclusion:**

The declining tendency in the density-independent population projection trajectories of the resistant chromosome-substituted lines could explain the simultaneous decline in the levels of resistance to the three organophosphates, observed in the Katsunuma population in the fall.

## Background

The development of insecticide resistance in insect pest populations is a population genetic process, in which insecticides select for initially rare resistant mutants within a population [1]. Although the development of insecticide resistance is an inevitable consequence of insecticide application, because the purpose of insecticide usage is to kill a certain portion of the insect pest population, insect populations that have developed resistance to insecticides sometimes exhibit reduction in levels of resistance to insecticides, after having been released from insecticide application. The cause of this reduction in resistance levels has been controversial [2]. Crow [1] said

"Since the genes causing insecticide resistance were at low frequency in the population before the insecticide began to be applied, it must ordinarily be true that they are to some extent disadvantageous; otherwise they would have been common. Therefore the selection for resistance should ordinarily involve the replacement of the original genes with *R *factors that, in every respect except insecticide resistance, are deleterious from a survival standpoint."

Under this theory, it could be expected that there is variation in fitness among resistant and susceptible genotypes, and that resistant genotypes have lower fitness than susceptible genotypes, which could result in the change of frequencies of resistance factors within a population.

The Katsunuma population (Yamanashi Prefecture, Japan) of *Drosophila melanogaster *is one of the well-established natural populations in Japan [3]. Katsunuma is famous for its vineyards, which extend continuously through the town, and its wine production. In the fall, masses of squeezed grapes are dumped outside during the process of wine production, and *Drosophila *flies drastically increase in number on them [4,5]. In this natural population, we have observed seasonal fluctuations in levels of resistance to three organophosphates for two consecutive years; susceptibility to the three organophosphates tended to increase in the fall [5]. To reveal the genetic basis of the seasonal fluctuations in susceptibility to the three organophosphates, we have compared several fitness measures among chromosome-substituted lines, whose chromosomes were derived from a resistant or a susceptible line collected before the population started expanding [6,7]. Lower fitness measures were generally obtained for resistant chromosome-substituted lines, which supports the previous hypothesis that resistant genotypes have lower fitness than susceptible genotypes.

In the Katsunuma population, the drastic change in food and breeding environments occurs from summer to fall. Almost density-independent conditions are suddenly created by masses of squeezed grapes. However, the period of these conditions might not be long enough for the population to attain a stable age-structure; rapid expansion and contraction occur during a rather short period of time. In fact, the Katsunuma population rapidly decreased in mid-November [7]. Therefore, it is also necessary to examine population trajectories during the phase before the population attained a stable age-structure. In this study, we further investigated density-independent population projection trajectories initiated from a single adult female for each chromosome-substituted line, after constructing a Leslie matrix for each line, which may reveal the responses of genotypes similar to each chromosome-substituted line to density-independent environments during a short period of time.

## Results

**Figure 1:**
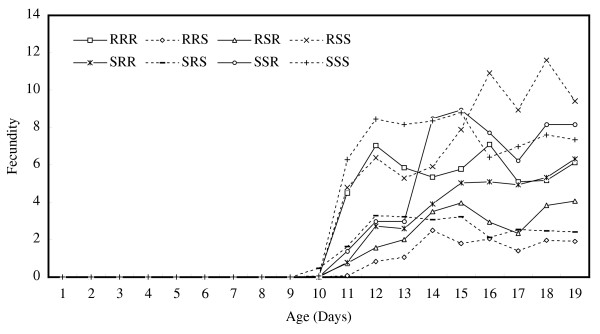
Age-specific fecundity for each chromosome-substituted line, averaged over replications. Each chromosome-substituted line is indicated by its chromosome composition (X, second and third chromosome in order). R and S indicate the origin of the chromosome: R for the resistant line #609-10 and S for the susceptible line #451-4. (solid line): resistant line; (dotted line): susceptible line.

**Figure 2:**
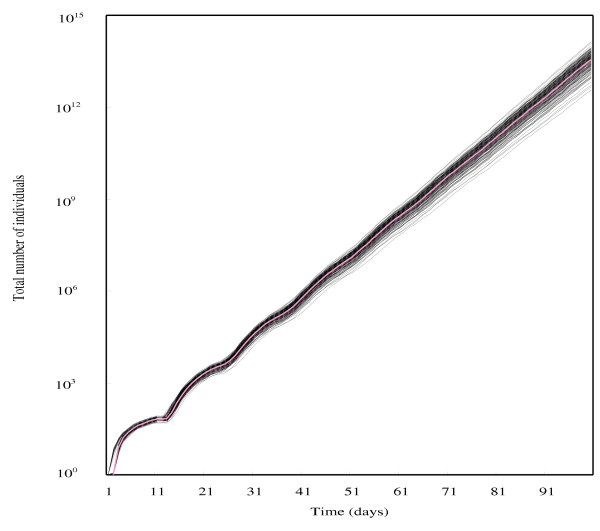
Density-independent population projection trajectories from a single adult female for line SSS. (red line): trajectory obtained from observed data; (black line): trajectory obtained from resampled data.

**Figure 3:**
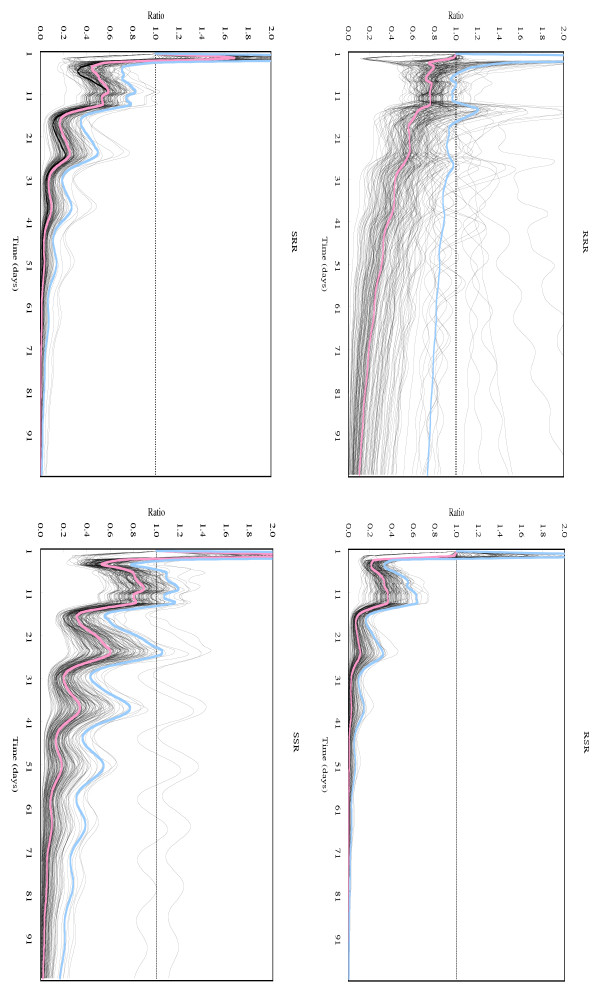
Density-independent population projection trajectories, expressed as ratio to line SSS, for resistant chromosome-substituted lines. Each chromosome-substituted line is indicated by its chromosome composition (X, second and third chromosome in order). R and S indicate the origin of the chromosome: R for the resistant line #609-10 and S for the susceptible line #451-4. (red line): trajectory obtained from observed data; (blue line): 95% upper confidence bound for the true population projection trajectory; (black line): trajectory obtained from resampled data.

**Figure 4:**
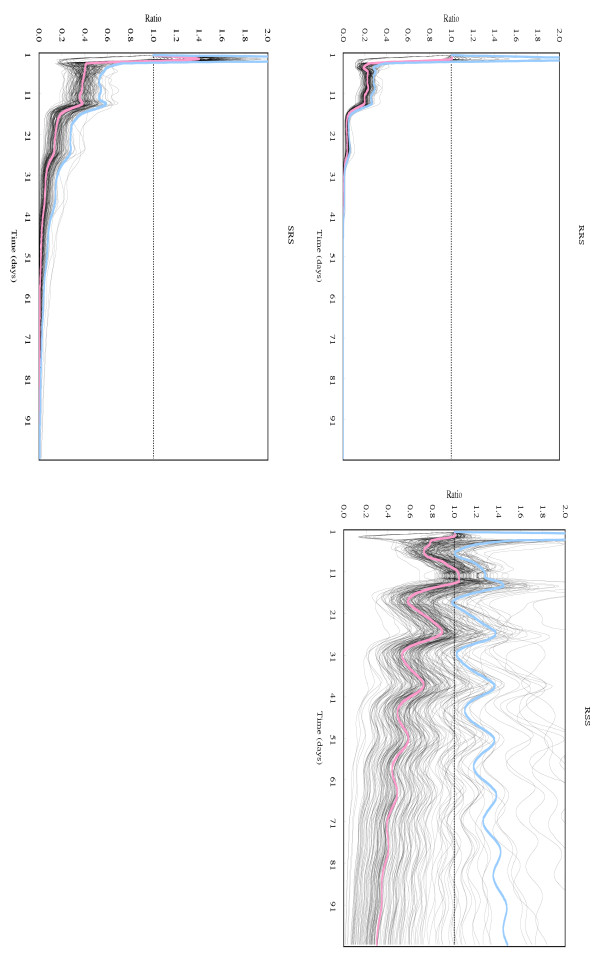
Density-independent population projection trajectories, expressed as ratio to line SSS, for susceptible chromosome-substituted lines. Each chromosome-substituted line is indicated by its chromosome composition (X, second and third chromosome in order). R and S indicate the origin of the chromosome: R for the resistant line #609-10 and S for the susceptible line #451-4. (red line): trajectory obtained from observed data; (blue line): 95% upper confidence bound for the true population projection trajectory; (black line): trajectory obtained from resampled data.

Age-specific fecundity averaged over replications, which were used for constructing a Leslie matrix for each chromosome-substituted line, is shown in Figure 1. Under the density-independent conditions in this study, a genotype similar to line SSS whose chromosomes were all derived from the susceptible line #451-4 could increase the number of individuals totaled over all age-classes to 1.0 × 10^7 ^on Day 50 and 3.6 × 10^13 ^on Day 100, starting from a single adult female (Fig. 2). Density-independent population projection trajectories for the other chromosome-substituted lines were expressed as the ratios of the total number of each line to that of line SSS. These are shown in Fig. 3 for resistant lines and in Fig. 4 for susceptible lines, respectively. Because a ratio of 1.0 (dotted lines in these figures) means that the number of individuals of the chromosome-substituted line is equal to that of line SSS, declining curves indicate that the relative number of individuals of that chromosome-substituted line is decreasing, compared to line SSS. All resistant lines showed decreasing ratios with fluctuations, compared to the total number of line SSS (Fig. 3). On Day 50, the ratio of the number of individuals to line SSS was 0.314 (0.848; 95 % upper confidence bound) for line RRR, 0.008 (0.066) for line RSR, 0.036 (0.142) for line SRR, and 0.181 (0.537) for line SSR. On Day 100, the ratio of the number of individuals to line SSS was 0.103 (0.736) for line RRR, 0.000 (0.005) for line RSR, 0.001 (0.014) for line SRR, and 0.022 (0.169) for line SSR. All susceptible lines also showed decreasing ratios with fluctuations, compared to line SSS, but the decline in the ratio was not significant in case of line RSS (Fig. 4). On Day 50, the ratio of the number of individuals to line SSS was 0.001 (0.002; 95 % upper confidence bound) for line RRS, 0.582 (1.345) for line RSS, and 0.018 (0.072) for line SRS. On Day 100, the ratio of the number of individuals to line SSS was 0.000 (0.000) for line RRS, 0.297 (1.479) for line RSS, and 0.000 (0.006) for line SRS.

**Figure 5:**
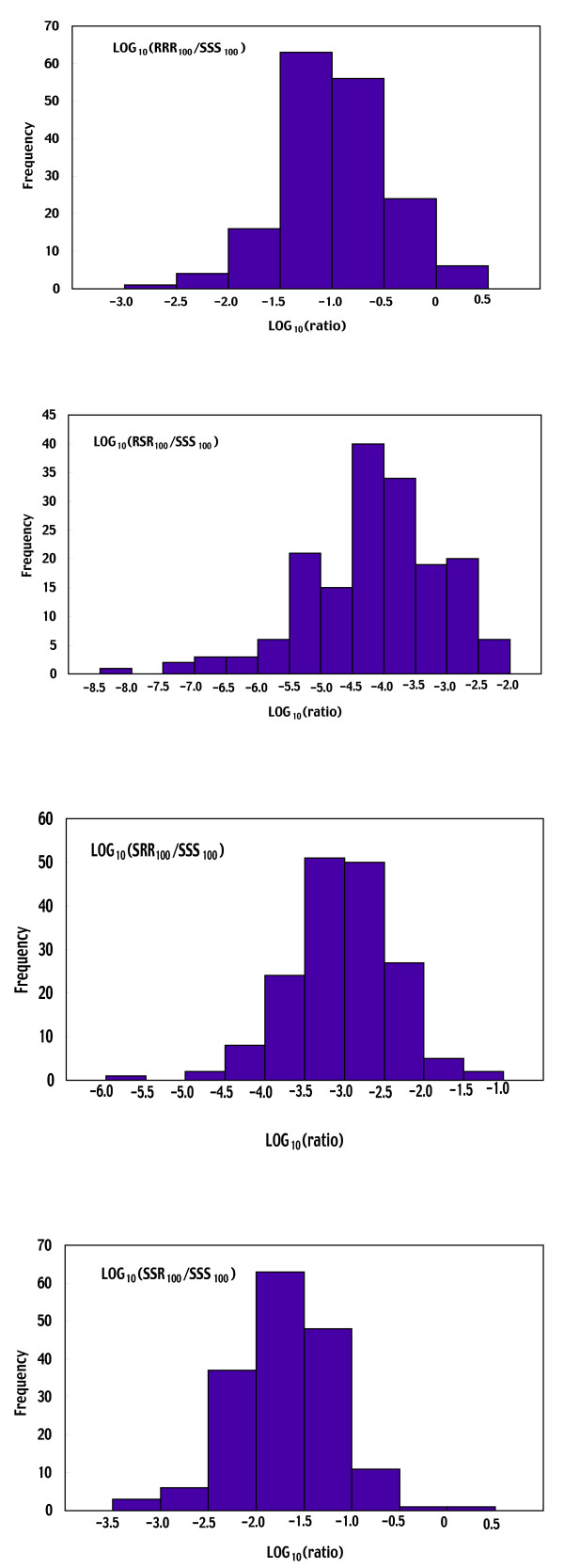
170 bootstrap replications of the log-transformed ratio of the total number of each resistant chromosome-substituted line to that of line SSS at Day 100. Each chromosome-substituted line is indicated by its chromosome composition (X, second and third chromosome in order). R and S indicate the origin of the chromosome: R for the resistant line #609-10 and S for the susceptible line #451-4.

**Figure 6:**
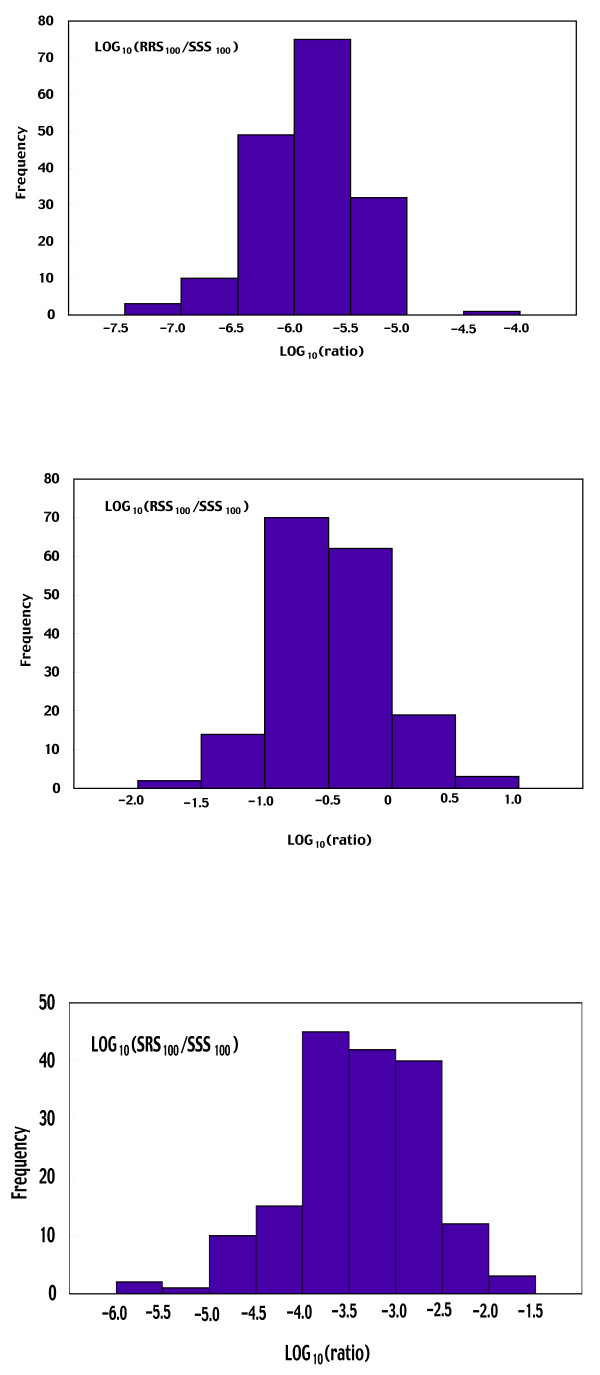
170 bootstrap replications of the log-transformed ratio of the total number of each susceptible chromosome-substituted line to that of line SSS at Day 100. Each chromosome-substituted line is indicated by its chromosome composition (X, second and third chromosome in order). R and S indicate the origin of the chromosome: R for the resistant line #609-10 and S for the susceptible line #451-4.

The distribution of 170 bootstrap replications of the log-transformed ratio of the total number of each line to that of line SSS on Day 100 is shown in Fig. 5 for resistant lines and in Fig. 6 for susceptible lines, respectively. These distributions seem reasonably normal. In fact, for each chromosome-substituted line, the log-transformed ratio obtained using the observed data, and mean and median of 170 bootstrap replications of the log-transformed ratio were very close (Table 1), suggesting that the log-transformed ratio obtained using the observed data was nearly unbiased for each chromosome-substituted line.

**Table 1:** log (ratio at Day 100) obtained from observed and resampled data.

	RRR	RRS	RSR	RSS	SRR	SRS	SSR
Observed	-0.987	-5.928	-4.103	-0.527	-2.941	-3.410	-1.652
Mean (Bootstrap replications)	-0.967	-5.881	-4.156	-0.463	-3.025	-3.396	-1.685
Median (Bootstrap replications)	-0.992	-5.872	-4.061	-0.511	-3.011	-3.378	-1.653

Each chromosome-substituted line is indicated by its chromosome composition (X, second and third chromosome in order). R and S indicate the origin of the chromosome: R for the resistant line #609-10 and S for the susceptible line #451-4.

## Discussion

### Population projection trajectories of chromosome-substituted lines

Chromosome-substituted lines, whose third chromosomes were derived from the resistant line #609-10, showed resistance to organophosphates [6]. In *D. melanogaster*, one of the major mechanisms for organophosphate resistance is caused by mutated acetylcholinesterase, the target site of organophosphate insecticides [8]. Mutations in acetylcholinesterase can change the catalytic activity and stability of this enzyme, which may lead to a fitness cost associated with organophosphate resistance [9]. In this study, all the resistant chromosome-substituted lines showed a significant tendency towards a decrease in the ratio of the total number of individuals to that of line SSS, under density independent conditions (Fig. 3). This was especially so for resistant line SSR, for which only the third chromosome was derived from the resistant line #609-10 and the other two major chromosomes were from the susceptible line #451-4. These results suggest that resistant genotypes tend to decrease in frequency under the density-independent conditions, even during the phase before the population attains a stable age-structure.

The calculations we have presented here do not, of course, relate to the changes in frequencies of genotypes in a sexually reproducing population. However, they should provide insight into whether resistance genes are likely to be prevented from spreading to fixation, since the initial rate of change in frequency of a rare gene in a randomly mating population will follow the dynamics that we have studied here ([10] pp. 149–166).

In the previous study, we compared the intrinsic rate of increase among the chromosome-substituted lines used in this study [7]. Resistant lines generally showed lower intrinsic rates than line SSS, but line RRR, whose chromosomes were all derived from the resistant line, had the intrinsic rate of increase that was not significantly different from that of line SSS [7]. Although some interactions among resistant chromosomes were suggested, we could not determine the role of the interactions in the seasonal fluctuations in susceptibility to three organophosphates. Because the decline of line RRR in the ratio was relatively slow, compared to the other resistant lines (Fig. 3), interactions among resistant chromosomes may prohibit rapid decline in the relative number of individuals of resistant genotypes. However, these interactions cannot prevent the decline in frequency of resistant lines under density-independent conditions before the population attains a stable age-structure. Therefore, these results suggest that the interactions among resistant chromosomes may not play a significant role in the seasonal fluctuations in susceptibility to the organophosphates within the Katsunuma population of *D. melanogaster*.

All the susceptible chromosome-substituted lines also showed a decline in this ratio with respect to line SSS, although the decline in line RSS was not significant (Fig. 4). Because these lines do not have resistance factor(s), these results obviously suggest that other factor(s), rather than the resistance factor(s), may affect the density-independent population projection trajectories of these lines during the phase before reaching a stable structure.

Relatively high frequencies of fitness-related mutations have been maintained in the Katsunuma population of *D. melanogaster *(viability [3,4]; fertility [3,11-13]; productivity [14]). The frequency of recessive lethal genes on the second chromosome in 1997 was estimated as 25% and that of male sterile genes was 10%, with fluctuations since early 1960s [3]. Because resistance factors were not located on the second chromosome of the resistant line #609-10, the declining tendencies in lines RRS and SRS were not correlated with the resistance factor(s). Therefore, it is possible that the resistant line #609-10 accidentally possessed the above fitness-related mutation(s) in the Katsunuma population on the second chromosome.

### Resistant genotype frequency in the Katsunuma population

Following the previous study, which introduced calculating the intrinsic rates of increase for the resistant and susceptible chromosome-substituted lines, this study sheds some more light on the genetic basis of the seasonal fluctuations in susceptibility to three organophosphate insecticides, observed within the Katsunuma population for two consecutive years [5]. Our results support the hypothesis that there is variation in fitness among resistant and susceptible genotypes. In particular, because the fitnesses of the resistant and susceptible chromosome-substituted lines were evaluated by the population projection trajectories generated by a single adult female during the phase before attaining a stable age-structure, which might represent a relatively short period of the density-independent conditions at Katsunuma, this study provide a further genetic basis for understanding the seasonal fluctuations in susceptibility to the three organophosphates from summer to fall.

Variation in fitness among resistant and susceptible genotypes could change the frequencies of the resistant genotypes in the fall Katsunuma population, where almost ampler density-independent conditions are suddenly created by masses of squeezed grapes dumped outside. Because the third chromosome of the resistant lines confers resistance to all of the three organophosphates [6], a significant tendency towards a decline in the ratio of each resistant chromosome-substituted line relative to line SSS could explain the simultaneous decline in the levels of resistance to the three organophosphates, observed in the Katsunuma population [5].

## Conclusions

In this study, we calculated population projection trajectories of chromosome-substituted lines resistant and susceptible to three organophosphate insecticides. All the resistant lines showed a significant tendency toward decrease in the numbers relative to line SSS. This study strongly supports the hypothesis that there is variation in fitness among resistant and susceptible genotypes, and that resistant genotypes have lower fitnesses than susceptible ones.

## Methods

### Drosophila lines

We obtained vital statistics from chromosome-substituted lines, whose chromosomes were derived from a resistant inbred line #609-10 and a susceptible inbred line #451-4, constructed by using a balancer stock, *w*; *Sp*/SM1; *Pr Dr*/TM3 [7,15]. Both of the lines were derived from the same natural population collected at Katsunuma on July 31, 1997, when the *D. melanogaster *population had not yet started expanding [16]. The resistant line #609-10 had low-to-moderate levels of resistance to three organophosphates, malathion, prothiophos and fenitrothion [16]. Although at least two resistance factors, one on the second chromosome and the other on the third chromosome, were shown to be involved in the Katsunuma population of *D. melanogaster *[15], the resistance factor(s) for all the three organophosphates were located on the third chromosome in the resistant line #609-10 [6]. Flies used for experiments were grown on glucose-yeast-cornmeal-agar medium in glass vials (3 cm in diameter and 10.5 cm in height) in incubators at 25 ± 0.5°C with a photoperiod of 14: 10 (L: D) h.

In this paper, each chromosome-substituted line was represented by its chromosome composition. For example, SRS indicates the chromosome-substituted line possessing the X and third chromosomes derived from the susceptible line #451-4 (S) and the second chromosome from the resistant line #609-10 (R).

### Population projection

**Figure 7:**
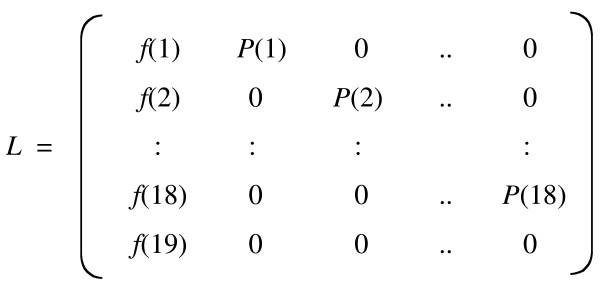
A 19 × 19 Leslie matrix, used for calculating density-independent population projection trajectories from a single adult female. Age-specific fecundity, *f*(*x*), and survival probabilities, *P*(*x*), over 19 days were used to construct a 19 × 19 Leslie matrix for each chromosome-substituted line.

Density-independent population projection trajectories from a single adult female were obtained for each chromosome-substituted line, after constructing a 19 × 19 Leslie matrix for each line (Fig. 7; cf. [17]).

A Leslie matrix consists of elements, which are age-specific fecundity (the first column in this matrix form, *f*(*x*)), age-specific survival probabilities (the off-diagonal elements, *P*(*x*)), and zero [10]. *P*(*x*) values lie between 0 and 1, whereas *f*(*x*) values are necessarily more than or equal to zero [18]. For age-specific fecundity *f*(*x*), some values are zero, depending on the reproductive schedule of the organisms concerned [18]. By multiplying a row vector *n*(*t*), whose components are numbers of each age-class, the number of each age-class at the next time unit, *n*(*t *+ 1), can be calculated as *n*(*t *+ 1) = *n*(*t*)*L *[10].

To construct a Leslie matrix, age-specific survival probabilities and fecundity were based on demographic data, collected for each individual adult fly (cf. [19,20]). These were the same data set used for calculating the intrinsic rate of increase for each chromosome-substituted line in the previous study [7]. One pair of adult flies that emerged within six hours was placed in a food vial and transferred into a new vial every day. When the flies were transferred, we checked whether the flies were alive or dead, and counted newly emerged adult flies every day. 16 pairs were prepared for each chromosome-substituted line. Half of the newly emerged adult flies on each day were considered in the estimates of age-specific fecundity in this study. Age-specific survival probability was assigned to be one when the transferred adult fly was alive, and zero when the fly was dead [19,20]. Because adult females were transferred, the age-specific survival probabilities from egg to pupal stages for the adult females were assigned to be one. Ages of the flies placed in the food vials were estimated as the duration from the entry of the adult flies to first emergence of their offspring. Because this length of immature stage varied among replications and among chromosome-substituted lines, ages of the adult flies were not necessarily assigned to be the same among them. Although the ages of adult females that produced no adult offspring cannot be assigned in this study, 14 out of the 16 females producing no adult offspring survived over the 19-day census period; therefore, age-specific survival probabilities for these females were assigned to be one. Two females producing no adult offspring, one RRS and one RSR female, died during the 19-day census period. These females were therefore excluded from the analysis, because the age-specific survival probabilities cannot be defined for these females.

In the previous study, age-specific fecundity and survival probabilities over 19 days, based on each individual, were used for calculating the intrinsic rate of increase for each replication. In this study, the age-specific fecundity and survival probabilities over 19 days were used to construct a 19 × 19 Leslie matrix (Fig. 7), after averaged over replications. Density-independent population trajectories from a single adult female, which just started reproduction, were then projected, using the 19 × 19 Leslie matrix.

### Statistical procedures

Density-independent population projection trajectories were estimated as the ratios of the number of each chromosome-substituted line, totaled over all age-classes, to that of line SSS for 100 days. 95% upper confidence bounds (one-sided; [21]) were set for the true population projection trajectories, after standard errors were obtained using the following bootstrap procedures [22]. For each bootstrap replication, 16 (15 for RRS and RSR) replications for each chromosome-substituted line were resampled with replacement from the observed data set, using a random number generator (Fortran 77 program). Age-specific fecundity and survival probabilities were averaged over resampled replications to construct a Leslie matrix for each line. After projecting the Leslie matrix for each line, the ratios of the total number of each line to that of line SSS for 100 days were estimated for each bootstrap replication. We collected 170 bootstrap replications for estimating standard errors for the ratios. Taking the distribution's shape into account, ratios were first log-transformed, and standard errors and 95 % upper confidence bounds were set for the log-transformed values. 95 % upper confidence bounds were then back-transformed into the normal scale.

## Authors' contributions

TM carried out data collection and analyses, and drafted the manuscript. BC contributed to the computer programming and provided the theoretical background. All authors read and approved the final manuscript.
